# EMG-EMG coherence during voluntary control of human standing tasks: a systematic scoping review

**DOI:** 10.3389/fnins.2023.1145751

**Published:** 2023-05-12

**Authors:** Eiji Yamanaka, Yuki Horiuchi, Ippei Nojima

**Affiliations:** ^1^Division of Physical Therapy, Shinshu University School of Health Sciences, Nagano, Japan; ^2^Department of Rehabilitation Medicine, Tokyo Bay Rehabilitation Hospital, Chiba, Japan

**Keywords:** EMG-EMG coherence, intra-muscular coherence, inter-muscular coherence, standing control, scoping review

## Abstract

**Background:**

Intra- or inter-muscular (EMG-EMG) coherence is a simple and non-invasive method for estimating central nervous system control during human standing tasks. Although this research area has developed, no systematic literature review has been conducted.

**Objectives:**

We aimed to map the current literature on EMG-EMG coherence during various standing tasks to identify the research gaps and summarize previous studies comparing EMG-EMG coherence between healthy young and elderly adults.

**Methods:**

Electronic databases (PubMed, Cochrane Library, and CINAHL) were searched for articles published from inception to December 2021. We incorporated studies that analyzed EMG-EMG coherence of the postural muscles in various standing tasks.

**Results:**

Finally, 25 articles fulfilled the inclusion criteria and involved 509 participants. Most participants were healthy young adults, while only one study included participants with medical conditions. There was some evidence that EMG-EMG coherence could identify differences in standing control between healthy young and elderly adults, although the methodology was highly heterogeneous.

**Conclusion:**

The present review indicates that EMG-EMG coherence may help elucidate changes in standing control with age. In future studies, this method should be used in participants with central nervous system disorders to understand better the characteristics of standing balance disabilities.

## Introduction

Bipedal standing is a posture exclusive to humans, and maintaining the standing balance is essential for daily living. However, the standing posture in humans is characterized by a narrow base of support and a high center of mass, making the posture biomechanically unstable. Further, functional decline with age increases postural sway (Masui et al., 2005; Roman-Liu, 2018). In addition, patients with neurological and musculoskeletal disorders have poor balance control (Tyson and Connell, 2009; de Lima et al., 2021) and are at high risk of falling (Weerdesteyn et al., 2008; Moutzouri et al., 2017). Therefore, to prevent falls, the neurophysiological mechanisms of standing balance control in healthy young adults and elderly adults, and individuals with the disease should be clarified.

Given the activities of daily living, the standing balance in various standing postures and sensory perturbations should be controlled. Because difficulty in standing varies with the size of the base of support, the flexible support of the central nervous system (CNS) in standing postures is often assumed (Watanabe et al., 2018a; Nandi et al., 2019). Furthermore, the difficulty in channeling the correct standing balance and the related neuromuscular activities varies depending on the available sensory inputs (Carver et al., 2006; Jeka et al., 2010). For example, more significant postural sway and specific neuromuscular activity while standing with eyes closed than with eyes open (Danna-Dos-Santos et al., 2015; Walker et al., 2020) has been reported. In addition, elderly adults (Nojima et al., 2018; Fujio and Takeuchi, 2021) and patients with stroke (de Haart et al., 2004) have a greater loss of standing balance in response to sensory disturbances than healthy young adults. Therefore, the mechanism of CNS controls of the postural muscles when the base of support and sensory information change should be investigated to elucidate human standing control.

Recently, intra- or inter-muscular (EMG-EMG) coherence was developed to investigate the control of the CNS during human standing tasks. The coherence analysis quantifies the strength of the common oscillation between two signals. Thus, EMG-EMG coherence quantifies the common oscillatory inputs to the motor neurons in the frequency band (Farmer et al., 1993), and can be conducted simply by measuring a pair of electromyography signals non-invasively. EMG-EMG coherence is analyzed from the auto-spectra of EMG signal x and y (P_xx_ and P_yy_), and the cross-spectra (P_xy_) in each frequency (f) with the Fourier transform. The coherence is estimated using the following equation.


$$\begin{array}{l} |C_{xy}\left(f\right){|}^{2}=\frac{\left|P_{xy}\left(f\right)\right|}{P_{xx}\left(f\right)P_{yy}\left(f\right)} \end{array}$$

Coherence takes the range 0–1, with greater values reflecting higher common inputs to the two motor neuron pools. Therefore, EMG-EMG coherence can provide insight into how the CNS may synchronize and regulate motor control among a combination of a number of muscles. Also, the coherence in each frequency band is generated by various brain areas and circuits (spinal, subcortical, or cortical). Thus, EMG-EMG coherence can provide an estimate of the neural circuits and the neural origin contributing to motor tasks. Delta-band coherence reflects synchronous oscillations in motor unit firing (Lowery et al., 2007) and is thought to reflect subcortical activity (Boonstra and Breakspear, 2012). Alpha-band coherence is associated with multiple factors (Grosse et al., 2002) that reflect subcortical inputs and may also have some cortical contributions (Boonstra and Breakspear, 2012; Obata et al., 2014). In addition, beta-band coherence is strongly related to corticospinal drive (Conway et al., 1995; Farmer, 1998; Grosse et al., 2002). In standing tasks, EMG-EMG coherence is significant in bilateral homologous and unilateral plantar flexor pairs in the delta-band and some alpha frequencies (Mochizuki et al., 2006; Boonstra et al., 2009; Obata et al., 2014; Watanabe et al., 2018a,b,c). Delta-to-alpha band coherence between antagonistic muscles is increased with increasing standing difficulty (Nandi et al., 2019; Nojima et al., 2020), such as with unilateral standing. In addition, beta-band coherence between synergistic muscles is increased in relatively complex standing tasks (Watanabe et al., 2018a,b; Nandi et al., 2019; Nojima et al., 2020). This suggests that cortical activity is increased to maintain standing balance. Thus, EMG-EMG coherence in standing tasks comprise various muscle pairs and frequency bands, and therefore the analytical methods used in previous studies should be integrated to provide a direction for future research.

Several studies reported that elderly adults have greater EMG-EMG coherence in the delta-to-alpha band in bipedal standing compared to young adults (Obata et al., 2014; Walker et al., 2020) and that they are less likely to change the beta coherence in response to challenging standing task (Watanabe et al., 2018b). Thus, EMG-EMG coherence could provide insight into the neuromuscular control associated with age-related deficits of standing balance. Furthermore, patients with CNS disorder decreased beta-band coherence on the paretic muscle pairs during gait, which was associated with clinical scales (Nielsen et al., 2008; Kitatani et al., 2016). Therefore, EMG-EMG coherence especially in the beta-band can also serve as an indicator of impaired motor control mechanisms in CNS disorders with lesions in the corticospinal tract. However, it is unclear how much evidence exists for EMG-EMG coherence during standing tasks in individuals with CNS lesions.

Studies investigating EMG-EMG coherence in human standing tasks started in the 2000's and have been validated in various individuals and tasks. EMG-EMG coherence analysis may reveal differences in standing controls between different individuals and tasks, but there is no consensus on this point of view. Given that this is a relatively new approach to identifying voluntary control of human standing, the purpose of this systematic scoping review was to map the current literature pertaining to the analysis of EMG-EMG coherence during various standing tasks in individuals and to identify the gaps in the research. In addition, several studies have accumulated showing that EMG-EMG coherence during standing tasks differs between young and elderly adults. These studies could contribute to revealing the neurophysiological mechanisms of age-related decline in standing balance; however, no literature has systematically integrated the available evidence. Therefore, this scoping review focused on summarizing previous studies evaluating the difference in EMG-EMG coherence between healthy young and elderly adults.

## Methods

This systematic scoping review of the literature was conducted according to the Preferred Reporting Items for Systematic reviews and Meta-Analyses extension for Scoping Reviews (PRISMA-ScR) check-list (Tricco et al., 2018) (Supplementary material 1).

### Eligibility criteria

The inclusion criteria were studies evaluating EMG-EMG coherence during voluntary control of various standing tasks in humans. There was no restriction on the publication year, and only English papers published till the search date were considered. The exclusion criteria for this study were as follows: (1) coherence analysis other than EMG-EMG, such as EEG-EMG, COP-EMG, etc., (2) coherence analysis of “involuntary” phenomena including tremors, spasms, and response to external stimuli, (3) coherence analysis during voluntary contractions that are not standing tasks, (4) conference abstracts, (5) studied in animals and *in vitro* studies. This review included studies that met the aforementioned criteria 1-3 and also included EMG-EMG coherence analysis during voluntary standing tasks.

### Information sources

We searched PubMed, Cochrane Library, and CINAHL for full-text articles. The end date of the study period was December 31, 2021. References were also searched manually by reviewing the reference lists of the included studies.

### Search strategy

A Participant/Concept/Context (PCC) model was used to build the criteria for the search of electronic databases. The PCC model comprised the Participant: “limited to humans,” Concept: “EMG-EMG coherence, intra-/inter-muscular coherence, standing tasks,” and Context: “both laboratory and clinical settings.” We combined the search terms [(coherence) OR (coherent)] AND [(electromyo^*^) OR (emg)] AND [(postur^*^) OR (stand^*^) OR (stance) OR (balance)] (Supplementary material 2) and searched in all fields. The search was conducted by one author (E.Y.), and the consistency of the search results was determined by another researcher (Y.H.).

### Study selection

The eligibility of the included studies was determined independently by two authors (E.Y. and Y.H.), first by reading the title and abstract for screening and then by reading the full text. During the screening process of titles and abstracts, we excluded studies that did not explicitly mention the inclusion of standing tasks. However, for studies that did not clearly specify EMG-EMG coherence analysis in the title and abstract screening process, we made full-text eligibility determinations instead of excluding them during the initial screening phase, assuming that such analysis was reported as a sub-outcome. In cases of disagreement between the two authors, the opinion of a third researcher (I.N.) was sought.

### Data charting

A data-charting form was jointly developed by the reviewers to determine the variables to be extracted. We used a table as a charting form because it was most frequently reported in previous scoping reviews (Miake-Lye et al., 2016). Data charting was performed by one author (E.Y.), and the research team verified whether the charted summaries were consistent with those of the included articles. The data charting included citations (authors and years), participants (number of participants and participant characteristics), study design (cross-sectional or longitudinal), standing tasks, methods of coherence analysis (muscular pair and frequency bands), and principal findings.

### Summary of evidence in healthy young and elderly adults

Since the purpose of this review was to clarify the differences in EMG-EMG coherence between healthy young and elderly adults during standing tasks, we summarized the results of studies that compared these groups. The studies included in this summary were those that were identified in the participant section during the data charting phase as included both young (20–40 years) and elderly or older (>60 years) groups. We listed the results of each study rather than synthesized the results, because each study involved different muscle pairs and frequency bands for the coherence analysis.

## Results

### Search results

**Figure 1:**
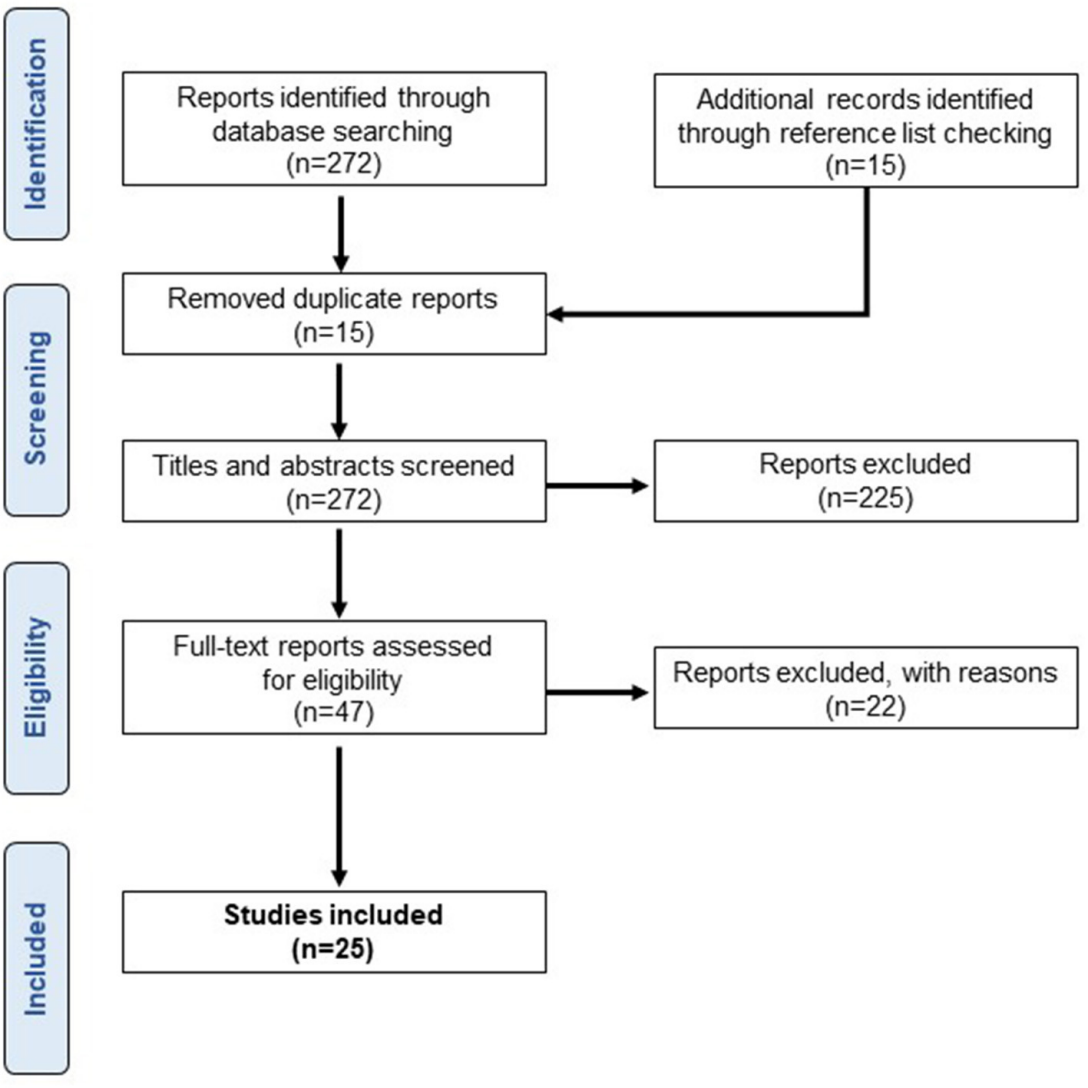
Study results. A flowchart of the selection process based on inclusion and exclusion criteria.

We obtained 272 articles from the database search and 15 articles from additional records. After removing duplicate articles (*n* = 15), the titles and abstracts of 272 publications were screened (Figure 1). Of these, 47 articles were assessed for eligibility by full-text screening, and 22 articles were excluded based on the following exclusion criteria: (1) coherence analysis other than EMG-EMG (*n* = 6), (2) coherence analysis of involuntary phenomena including tremors, spasms, and response to external stimuli (*n* = 9), (3) coherence analysis during voluntary contractions that are not standing tasks (*n* = 5), and (4) conference abstracts (*n* = 2).

### Characteristics of included studies

The characteristics of included studies are listed in Table 1. Of the 25 included studies, 21 were cross-sectional and only four were longitudinal studies. In these cross-sectional studies, the research purpose was mainly to compare EMG-EMG coherence among different participant groups and/or different standing tasks. Three longitudinal studies examined EMG-EMG coherence before and immediately after a single session of a fatigue protocol (Watanabe et al., 2018c) and alcohol intake (Boonstra et al., 2008, 2009), and the other longitudinal study investigated coherence before and after 14 weeks of resistance training (Walker et al., 2020).

**Table 1:** Summary of included studies in the review.

**Study**	**Participants**	**Standing tasks**	**Coherence**	**Principal findings**
Mochizuki et al. (2006)	−11 healthy young [28 (6) y]	- Bipedal standing - Isometric contraction	Pair: bilateral and unilateral SL-SL Frequency: 0–30 Hz	- In the postural task, bilateral and unilateral coherence was greater than in the voluntary task in the delta-band.
Obata et al. (2014)	−23 healthy young [23–35 y] - 21 healthy children [6–8 y] - 22 healthy elderly [60–80 y]	- Bipedal standing with EO - Bipedal standing with EC	Pair: bilateral SL, MG, unilateral SL-MG Frequency: 0–4, 8–12 Hz	- At alpha-band, only the elderly adults showed significant coherence. - At delta-band, the coherence for all muscle pairs was greater in the elderly group than in the young group.
Danna-Dos-Santos et al. (2015)	−9 healthy young adults [26 (3) y]	- Bipedal standing with EO - Bipedal standing with EC	Pair: M-mode: SL, BF, ES, TA, RF, RA Frequency: 0–55 Hz (1–10 Hz)	- Coherence for the posterior and anterior pairs was significant in the delta-to-alpha band during the EO and decreased in the EC.
Mohr et al. (2015)	−16 healthy young adults [26 (5) y]	- Bipedal squat - Unipedal squat - Isometric squat - Unipedal standing	Pair: unilateral VM-VL Frequency: 30–60 Hz	- Coherence was greater during bipedal, unipedal, and isometric squat but not unipedal standing compared to the reference.
García-Massó et al. (2016)	−20 healthy young adults [23 (5) y]	- Bipedal standing - Unipedal standing	Pair: M-mode: MG, BF, ES, TA, VM, RA, EO Frequency: 0–55 Hz (0–5 Hz)	- Coherence for all M-mode pairs was significant in the delta-band. - Mixed coherence was greater for unipedal than bipedal, and posterior coherence was greater for bipedal than unipedal standing.
Noé et al. (2017)	−18 healthy young adults [20 (3) y]	- STA -Bipedal - Bipedal with EC - AP -Bipedal on a seesaw device - Bipedal with EC on a seesaw device	Pair: unilateral pair SL, MG, TA, VM, BF, GM, ES, RA Frequency: 0–55 Hz	- In the AP condition, coherence was observed for TA-SL, TA-RA, MG-TA. - ES-SL, ES-MG, ES-TA coherence were in the AP with EO and decreased in EC. - The coherence for SL-RA was observed with EC but not with EO in both tasks.
Watanabe et al. (2018a)	−14 healthy young adults [23 (1) y] - 17 healthy elderly adults [70 (3) y]	- Bipedal standing - Unipedal standing	Pair: unilateral MG-LG, MG-SL, LG-SL Frequency: 0–5, 15–35 Hz	- In the delta, MG-SL was larger than others for the elderly and unipedal for the young. - In the delta-band, coherence was greater in the elderly than young adults in both tasks. - Beta band coherence was greater in unipedal than bipedal for both groups.
Watanabe et al. (2018b)	−14 healthy young adults [23 (1) y] - 19 healthy elderly adults [70 (3) y]	- Bipedal standing - Bipedal forward-leaning standing to 35 and 75% of the maximal lean	Pair: bilateral MG-MG, SL-SL, unilateral MG-SL Frequency: 0–5, 15–35 Hz	- In the delta-band, coherence was lower in the forward lean than quiet standing for bilateral MG and SL for young adults. - In beta, MG-SL coherence was larger in forward lean than quiet stance for young, no difference was observed for elderly.
Watanabe et al. (2018c)	−13 healthy young adults [22 (1) y]	- Bipedal standing before and after a fatigue (exhaustive heel raise)	Pair: bilateral and unilateral MG, SL Frequency: 0–5, 5–15, 15–35Hz	- Bilateral coherence in the delta- and beta-band decreased after the fatigue. - Unilateral coherence in the delta-band tended to decrease after the fatigue.
Nandi et al. (2019)	−20 healthy young adults [21 (1) y]	- Bipedal standing (wide base) - Bipedal standing (narrow base) - Tandem stance - Unipedal standing	Pair: unilateral SL-LG, SL-PL, LG-PL, SL-TA, LG-TA, PL-TA, RF-BF Frequency: 0–5, 6–15, 16–40 Hz	- In delta, agonist coherence was larger than antagonist in bipedal. Antagonist coherence was larger in unipedal than wide. - In the beta-band, agonist coherence was greater in unipedal than wide. The agonist coherence was greater than the antagonist.
Formaggio et al. (2019)	−14 Parkinson's disease with Pisa syndrome [73 (7) y]	- Sitting task - Standing task	Pair: thoracic and lumbar paraspinal muscles, OE Frequency: 0-25 Hz	- Coherence for all muscle groups remained below 0.3 in the frequency bands, which indicated a lack of coherence.
Walker et al. (2020)	−16 healthy young adults [18–31 y] - 17 healthy elderly adults [66–73 y]	- Bipedal standing - Bipedal standing with EC before and after the strength training (14-wk)	Pair: unilateral TA-SL, TA-MG, MG-SL Frequency: 2-6, 8-14, 16-30, 40-60 Hz	- In young, TA-MG, SL coherence increased from EO to EC. This was not in the elderly. - Strength-training did not alter the coherence changes from EO to EC task. - Coherence for SL-TA in beta-band with EC decreased in young after training.
Degani et al. (2020)	−10 healthy young adults [27 (3) y] - 10 healthy elderly adults [69 (4) y]	- Bipedal standing with EO - Bipedal standing with EC	Pair: M-mode: TA, RF, RA, SL, BF, ES Frequency: 0-55 Hz	- Coherence was observed for anterior, posterior, antagonist in the delta-to-alpha band, and was greater for the elderly group. - In delta-alpha, coherence for anterior and posterior decreased in young with EC, but no such change was observed in the elderly.
Nojima et al. (2020)	−43 healthy elderly adults [73 (7) y]	- Bipedal standing - Unipedal standing	Pair: unilateral TA, MG, LG Frequency: 0–5, 15–30 Hz	- In delta and beta band, MG-LG coherence was greater in unipedal than in bipedal. - In the beta-band, MG-LG coherence was associated with muscle mass.
Boonstra et al. (2009)	−10 healthy young adults (21–30 y)	- Bipedal standing (forward and backward COP tracking task) before and after drinking alcohol	Pair: bilateral SL-SL, MG-MG, TA-TA, RF-RF, BF-BF Frequency: 6–11, 29–34 Hz	- Bilateral coherence was in lower leg pairs and largely absent between upper leg pairs. - Coherence decreased for SL and MG in alpha-band with alcohol ingestion. - Coherence was not constant in time, but modulated within the COP movement.
Danna-Dos-Santos et al. (2014)	−9 healthy young adults [29 (6) y]	- Bipedal standing with upper limbs raised with 5 kg barbell	Pair: SL-BF, SL-ES, BF-ES Frequency: 0–5, 5–20, 20–55 Hz	- Coherence was observed for the delta-alpha, and greater than beta-gamma band. - SL-BF coherence was greater than BF-ES and SL-ES.
von Tscharner et al. (2014)	−13 healthy young adults [recreational athlete; 20–30 y]	- Heel raise task in the edge of a step - Tiptoe position (calf rising)	Pair: four proximal and distal MG Frequency: 25–400 Hz	- The coherence in the tiptoe position was lower compared to the heel rising task. - In both tasks, significant coherence was observed in 25 to about 200 Hz.
Tanabe et al. (2017)	−7 non-professional ballet dancers [24 (5) y]	- Bipedal standing with 6 leg positions, commonly used in ballet dance	Pair: unilateral GM, RF, SR, VL, BF, SM, MG, LG, SL, PL, TA, ED, FH Frequency: 2–50 Hz	- Ankle-knee coordination was associated with coherence for LG and other muscle pairs, while knee-hip coordination was associated with the coherence for the RF and SR.
Degani et al. (2017)	−9 healthy young adults [29 (6) y] - 13 healthy elderly adults [69 (3) y]	- Bipedal standing with upper limbs raised with 5 kg barbell	Pair: unilateral SL-BF, SL-ES, BF-ES Frequency: 0–4, 0–10, 10–15 Hz	- Young showed significant coherence for SL-BF in delta-band, whereas elderly showed within delta-to-alpha band. - Elderly had greater coherence than the young in the delta-to-alpha band.
Kenville et al. (2020)	−11 healthy young adults [28 (5) y]	- Bipedal squatting (ECC, ISO, and CON)	Pair: bilateral ES, VL, VM, TA Frequency: 8-12, 13-30, 30-44 Hz	- Coherence was larger in ECC and ISO for ES than almost other pairs in alpha-band. - In beta, ES coherence was greater than the other, and greater for TA than VL and VM. - In gamma, ES was larger than TA and VE.
Hug et al. (2021)	−18 healthy young adults [29 (8) y]	- Heel raise task (neutral, toe-in) - Isometric contraction	Pair: unilateral, within MG, LG, SL Frequency: 0-5, 5-15, 15-35 Hz	- The proportion of common synaptic input to the intramuscular pair was lower in SL - Overall, minimal unilateral coherence was observed, regardless of the frequency.
Minamisawa et al. (2021)	−16 healthy young [20 (1) y] - 18 healthy elderly[73 (4) y]	- Bipedal standing	Pair: contralateral, unilateral, and bilateral SL, MG Frequency: 0-10 Hz	- In elderly, all coherence exist in delta-alpha, and those of young were in delta. - The correlation between the postural sway and coherence was moderate in the elderly.
Boonstra et al. (2016)	−18 healthy young adults [26 (5) y]	- Bipedal standing - With counting backward - Holding a cup - At a height of 1 m	Pair: bilateral and unilateral RF, VM, MG, TA, ED Frequency: 0–60 Hz	- Coherence was larger for lower leg and same segments in broad range of frequency. - In the height, coherence was greater. - In counting, coherence increased in alpha. - Coherence decreased in holding a cup.
Saffer et al. (2008)	−10 healthy young adults [19–32 y]	- Bipedal standing - Bipedal standing with EC	Pair: unilateral LG, SL, TA, BF, RF, RA, ES Frequency: 0.05–5 Hz	- Significant coherence was observed only between the LG-SL, and RF-SL. - No difference was observed in coherence for visual conditions.
Boonstra et al. (2008)	−10 healthy young adults [19–23 y]	- Bipedal standing - Bipedal standing with EC before and after drinking alcohol	Pair: bilateral LG, MG, SL, TA, ED Frequency: 0–5, 10–15 Hz	- Flexor coherence was absent, and those for extensors were observed in delta-alpha. - Alcohol decreased alpha coherence. - The coherence for extensors increased in both frequency bands with EC.

EO, eyes-opened; EC, eyes-closed; TA, tibialis anterior; MG, medial gastrocnemius; LG, lateral gastrocnemius; SL, soleus; BF, biceps femoris; ES, erector spinae; RF, rectus femoris; RA, rectus abdominus; VM, vastus medialis; VL, vastus lateralis; PL, peroneus longus; OE, obliquus externus; GM, gluteus medius; SR, Sartorius; FH, flexor hallucis brevis; ED, extensor digitorum longus.

The 25 included studies involved 509 participants, including 315 healthy young adults, 159 healthy elderly adults, 21 healthy children, and 14 patients with idiopathic Parkinson's disease and Pisa syndrome. Sixteen studies (Mochizuki et al., 2006; Boonstra et al., 2008, 2009, 2016; Saffer et al., 2008; Danna-Dos-Santos et al., 2014, 2015; von Tscharner et al., 2014; Mohr et al., 2015; García-Massó et al., 2016; Noé et al., 2017; Tanabe et al., 2017; Watanabe et al., 2018c; Nandi et al., 2019; Kenville et al., 2020; Hug et al., 2021) involved a single group of healthy young adults, of which, one study involved a group of non-professional classical ballet dancers (Tanabe et al., 2017). Only one study (Nojima et al., 2020) involved a single group of healthy elderly adults. Six studies (Degani et al., 2017, 2020; Watanabe et al., 2018a,b; Walker et al., 2020; Minamisawa et al., 2021) analyzed two groups of healthy young and elderly adults, and one study (Obata et al., 2014) assessed three groups of healthy young adults, elderly adults, and children. Only one study (Formaggio et al., 2019) recruited participants with physical disabilities and Parkinson's disease with Pisa syndrome. While several studies investigated healthy young and elderly adults, the results for patients in clinical settings were revealed only to a limited extent.

### Standing tasks

Various standing tasks were performed in previous studies (Table 1). Most studies (Mochizuki et al., 2006; Boonstra et al., 2008, 2016; Saffer et al., 2008; Obata et al., 2014; Danna-Dos-Santos et al., 2015; García-Massó et al., 2016; Noé et al., 2017; Watanabe et al., 2018a,b,c; Formaggio et al., 2019; Nandi et al., 2019; Degani et al., 2020; Nojima et al., 2020; Walker et al., 2020; Minamisawa et al., 2021) reported common tasks as natural bipedal standing. In addition, several studies implemented unipedal (García-Massó et al., 2016; Watanabe et al., 2018a; Nandi et al., 2019; Nojima et al., 2020) or bipedal standing with eyes closed (Saffer et al., 2008; Obata et al., 2014; Danna-Dos-Santos et al., 2015; Noé et al., 2017; Degani et al., 2020; Walker et al., 2020), and compared with natural bipedal standing. Other standing tasks included shifting the center of pressure within the base of support (Boonstra et al., 2009; Watanabe et al., 2018b), dual tasks (Boonstra et al., 2016), standing at a height (Boonstra et al., 2016), squatting (Mohr et al., 2015; Kenville et al., 2020), and heel raises (von Tscharner et al., 2014; Hug et al., 2021). EMG-EMG coherence increased as the base of support narrowed and the difficulty in standing increased (Watanabe et al., 2018a; Nandi et al., 2019; Nojima et al., 2020). These studies reported that beta-band coherence, in particular, varied with the complexity of the standing tasks. Regarding comparisons with and without visual information, no clear consensus can be reached. Several studies (Boonstra et al., 2008; Obata et al., 2014; Walker et al., 2020) reported increased coherence during eye-closed conditions than during natural standing, but a few studies found no change (Saffer et al., 2008) or the opposite effect (Danna-Dos-Santos et al., 2015; Degani et al., 2020).

### EMG-EMG coherence analysis

**Figure 2:**
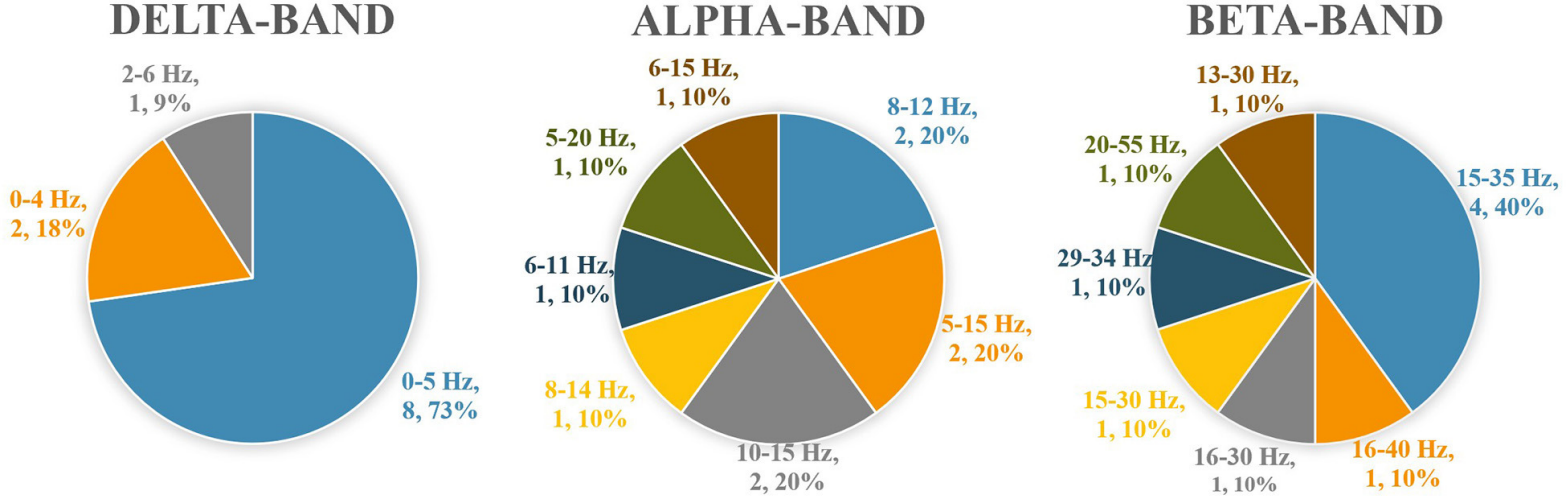
Numerical values separating the delta, alpha, and beta bands applied in the included studies.

Coherence analyses were performed in intra-muscular (von Tscharner et al., 2014; Hug et al., 2021), bilateral homonymous muscle pairs (Mochizuki et al., 2006; Boonstra et al., 2008, 2009, 2016; Obata et al., 2014; Watanabe et al., 2018b,c; Kenville et al., 2020; Minamisawa et al., 2021), unilateral plantar flexor pairs (Mochizuki et al., 2006; Saffer et al., 2008; Obata et al., 2014; Noé et al., 2017; Watanabe et al., 2018a,b,c; Nandi et al., 2019; Nojima et al., 2020; Walker et al., 2020; Hug et al., 2021; Minamisawa et al., 2021), unilateral knee extensor pairs (Mohr et al., 2015; Boonstra et al., 2016; Tanabe et al., 2017), and unilateral antagonistic muscle pairs (Saffer et al., 2008; Boonstra et al., 2016; García-Massó et al., 2016; Noé et al., 2017; Tanabe et al., 2017; Nandi et al., 2019; Degani et al., 2020; Nojima et al., 2020; Walker et al., 2020). Several studies (Danna-Dos-Santos et al., 2015; García-Massó et al., 2016; Degani et al., 2020) investigated muscle pairs across joints according to the components of muscle synergy (M-mode). There was no standard set of muscle pairs for analysis in the standing tasks, and the variability between the studies was high (Table 2). The delta-band was the most frequent frequency band of interest for coherence analysis. Many studies quantified coherence by classifying it into delta, alpha, beta, and gamma bands. However, the specific values for classifying the delta, alpha, and beta bands were researcher-dependent and differed among the studies. Most studies selected 0–5 Hz for the coherence analysis of the delta-band, and about half of the studies selected 15–35 Hz for the beta-band (Figure 2).

**Table 2:** Muscle pairs and frequency reported in the included studies.

		**Delta-band**	**Alpha-band**	**Beta-band**
Bilateral pair	SL-SL	◦◦◦◦◦◦ •• ■	◦◦◦◦ •• ■	■
	MG-MG	◦◦◦◦◦ •• ■	◦◦◦◦ •• ■	■
	TA-TA	◦ •	◦ • ■	■
	RF-RF	◦	■	■
	BF-BF		■	■
	ED-ED	◦ •	◦ •	
	LG-LG	◦ •	◦ •	
Unilateral pair	SL-MG	◦◦◦◦◦ •• □	◦◦◦◦ ••	◦◦◦◦ • □■
	SL-LG	◦◦◦ • □	◦□	◦◦□□
	SL-PL	◦□	◦□	◦□
	MG-LG	◦◦□□		◦◦□□
	LG-PL	◦□	◦□	◦□
	TA-SL	◦◦ • □	◦◦ • □	◦◦ • □
	TA-MG	◦◦ • □	◦ •	◦◦ • □
	TA-LG	◦◦□□	◦□	◦◦□□
	TA-PL	◦□	◦□	◦□
	SL-RF	◦ •		
	RF-BF	◦□	◦□	◦□
	M-mode	◦◦ •• □	◦◦ ••	
Contralateral pair	SL-MG	◦	◦	

This table presents the frequency bands and muscle pairs reported in the EMG-EMG coherence analysis across the studies included. White circles indicate bipedal quiet standing with eyes-opened, black circles indicate bipedal standing with eyes-closed, white squares denote unipedal standing, and black squares represent dynamic standing tasks that involve center of pressure displacement within the base of support. Any other standing tasks or studies that performed coherence analysis across a broad bandwidth (e.g., 0–55 Hz) without restricting the frequency bands are not included in this table. SL, soleus; MG, medial gastrocnemius; TA, tibialis anterior; RF, rectus femoris; BF, biceps femoris; ED, extensor digitorum longus; LG, lateral gastrocnemius; PL, peroneus longus.

### Summary of studies comparing healthy young and elderly adults

Obata et al. (2014) reported that a significant coherence of 0–4 Hz was found for both groups in all muscle pairs (bilateral and unilateral plantar flexors), while a significant coherence of 8–12 Hz was found only for the elderly group in bilateral muscle pairs. Furthermore, coherence of 0–4 and 8–12 Hz were higher in the elderly group, and in the closed-eye condition.

Watanabe et al. (2018a) also found that during bipedal standing, the delta-band coherence of the plantar flexor pairs was greater in elderly adults than in young adults. Similarly, the delta-band coherence for the plantar flexor pair was greater in unipedal than in bipedal standing for both age groups and in the elderly than in young adults. In addition, the beta-band coherence for this pair was greater than the other pairs in unipedal standing for elderly adults, which implies that elderly adults have selectively increased corticospinal drive to these muscles to cope with postural sway. Another study (Watanabe et al., 2018b) allotted quiet standing and standing tasks in which the center of pressure was maintained in front of the base of support. When the center of pressure was moved forward, the beta-band coherence of the unilateral plantar flexor pairs increased in young adults, whereas no such change was observed in elderly adults.

Minamisawa et al. (2021) reported that during bipedal standing, significant coherence below 10 Hz was observed in bilateral and unilateral plantar flexor pairs in the elderly group, whereas significant coherence was below 4 Hz in the young group. They also found that the correlation coefficient between the center of mass acceleration and coherence level was low in young adults and moderate in elderly adults. These results suggest that EMG-EMG coherence is related to increased postural sway during standing with age.

Walker et al. (2020) examined changes in coherence after 14 weeks of resistance training in open- and closed-eye standing positions. Before the training, apparent increasing of the medial gastrocnemius and tibialis anterior coherence levels was observed in closed-eye standing tasks compared to the open-eye standing tasks in young adults, which was not observed in elderly adults. In addition, strength training did not alter the significant coherence increase between open- and closed-eyes standing conditions in either muscle pair. The only training-induced change in coherence magnitude was observed in the soleus and tibialis anterior pair for the beta-band during the closed-eye standing task, which was lower in young adults after training.

Degani et al. (2017) reported EMG-EMG coherence in the pairs of synergistic muscle groups. They found that young adults presented with significant coherence of 0–5 Hz in the soleus and biceps femoris pair, whereas elderly adults presented with significant coherence of 0–10 Hz. In addition, elderly adults exhibited significantly greater coherence of 0–10 Hz in the soleus and elector spinae and biceps femoris and elector spinae pairs than young adults. These findings were consistent with the results of previous studies (Obata et al., 2014; Watanabe et al., 2018a,b). Another study (Degani et al., 2020) investigated EMG-EMG coherence during open- and closed-eyes bipedal standing with the M-mode. They found that young adults exhibited a significantly decreased coherence of 1–10 Hz in the anterior and posterior muscle pairs with eyes closed, while no such change was observed in elderly adults. This result was different from those of studies (Obata et al., 2014; Walker et al., 2020) that investigated the same standing tasks.

## Discussion

This scoping review was conducted to map the literature on EMG-EMG coherence during standing tasks in humans to identify preliminary findings and areas requiring further research. We identified that most studies were cross-sectional, and that more than half of the participants were healthy young adults. While various studies included healthy elderly adults for comparison with young adults, only one study has included participants with preexisting medical conditions. Patients with CNS disorders exhibited EMG-EMG coherence characteristics different from those of healthy participants in motor tasks other than standing tasks, and coherence was associated with severity of the disorder (Nielsen et al., 2008; Fisher et al., 2012; Kitatani et al., 2016). EMG-EMG coherence analysis could be easy to perform and helps to quantify neuromuscular activity during motor tasks non-invasively. Further research should be conducted on standing tasks better to understand the characteristics of the disability of standing balance.

Half of the previous studies included in this review analyzed natural bipedal standing, though other standing tasks were also analyzed. Most studies compared EMG-EMG coherence in multiple standing tasks and many employed standing tasks with varying sizes of the base of support and sensory perturbation. These studies reported different muscle pairs, frequency bands, and magnitudes in EMG-EMG coherence among standing tasks, suggesting that human standing flexibly modulates neuromuscular activity according to task characteristics. Interestingly, although healthy young adults showed significant changes in EMG-EMG coherence in several standing conditions, such as unilateral standing (Watanabe et al., 2018a), forward leaning (Watanabe et al., 2018b), and sensory perturbation (Degani et al., 2020; Walker et al., 2020), these changes were limited in healthy elderly adults. These showed the importance of including multiple standing tasks and analyzing the changes in coherence between standing tasks in this research area. Thus, more challenging tasks could be analyzed in addition to bipedal standing to elucidate the neural mechanisms associated with standing disturbance.

Many muscle pairs and frequency bands were investigated using the coherence analytical method, resulting in a high degree of heterogeneity among the studies. The most frequently assessed muscle pairs were the unilateral and bilateral plantar flexor pairs. In contrast, the muscle pairs that produce significant coherence vary with the difficulty and characteristics of the standing task. Thus, the muscle pairs to be investigated should be determined by the characteristics of the standing task. During quiet standing, numerous studies have shown significant coherence between bilateral and unilateral plantar flexor muscles (Table 2), making these muscle pairs the primary focus in this field of research. Furthermore, it is recommended to analyze antagonistic muscle pairs (i.e., unilateral tibialis anterior and plantar flexors) as co-contraction between agonist-antagonist muscles is likely to occur in challenging or fearful standing tasks, or in groups at high risk of fall (Boonstra et al., 2016; Nandi et al., 2019; Nojima et al., 2020). Regarding the frequency band in coherence analysis, significant coherence was observed primarily in the delta band, regardless of the muscle pair. The delta frequency band is thought to reflect the synchronous activity of the motor neuron pool (Lowery et al., 2007). In addition, several studies reported significant beta-band coherence in some standing tasks. Beta-band coherence is thought to reflect activity in the corticospinal tract (Conway et al., 1995; Farmer, 1998; Grosse et al., 2002), suggesting control from the cortex during standing tasks. Since significant beta-band coherence was reported in standing tasks that are characterized by a narrow base of support (i.e., unipedal standing) (Watanabe et al., 2018a; Nandi et al., 2019; Nojima et al., 2020) and forward leaning (Watanabe et al., 2018b), cortical control of standing may increase as the difficulty of the standing task increases. Patients with CNS disorders that damage the corticospinal tracts may not be able to control complex standing tasks, possibly because of the lack of beta-band coherence. This point of view must be further verified. Many studies have divided the EMG-EMG coherence into the delta, alpha, beta bands and quantified it in each band. However, the present review revealed that the specific values for classifying the delta, alpha, and beta bands were researcher-dependent and differed among the studies. Most studies selected 0–5 Hz for the coherence analysis of the delta-band, and about half of the studies selected 15–35 Hz for the beta-band. The authors would recommend selecting these bandwidths in the delta and beta coherence analysis in order to reduce heterogeneity and accumulate results in this research area. In contrast, no specific recommendations could be established for the alpha-band. This is probably related to the fact that coherence in these bands occurs multifactorial and is less likely to be as significant as in the delta-band coherence. A consistent method to set the parameters of coherence analysis for integrating the results in this research area should be determined in the future.

This scoping review did not synthesize the results, summarized each study to determine the heterogeneity. Despite these limitations, each study reported consistent trends between healthy young and elderly adults. For example, in natural bipedal standing, elderly adults reported higher coherence levels in some muscle pairs and significant coherence for broader frequency bands than young adults (Obata et al., 2014; Degani et al., 2017, 2020; Watanabe et al., 2018a,b; Walker et al., 2020; Minamisawa et al., 2021). This phenomenon could occur because elderly adults have more significant postural sway and require more descending neural input in the motor neuron pools, even in natural bipedal standing. Furthermore, EMG-EMG coherence analysis indicates neural oscillations from different circuits in each frequency hand, which implies that older adults may have a wider range of neural activity during standing control compared to younger adults. Conversely, higher coherence levels indicate greater common input to the two motor neuron pools, but this does not necessarily indicate a greater range of neural activity. Instead, coherence analysis provides information about the degree of coordination between different neural circuits in each frequency band, which may be related to the level of corticospinal activity. Although the main origin of alpha-band coherence is proposed to be the reticulospinal pathway (Obata et al., 2014), the corticospinal pathway may also contribute to coherence in this frequency band, given that corticomuscular coherence at approximately 10 Hz has been reported in previous study (Conway et al., 1995). Consequently, it is plausible that elderly adults may improve postural sway by increasing corticospinal tract activity during quiet standing. Thus, postural sway was reported to be associated with coherence levels in elderly adults (Watanabe et al., 2018a; Minamisawa et al., 2021). Previous study (Nojima et al., 2020) reported that increased beta-band coherence in elderly adults was associated with smaller muscle mass in lower leg. This suggests that the elderly adults may compensate for the loss of muscle mass by increasing activity in the corticospinal tract during standing tasks. It was also shown that elderly adults were less likely to experience changes in EMG-EMG coherence in response to changes in standing difficulty and characteristics (Watanabe et al., 2018b; Walker et al., 2020). This corresponds to the fact that elderly adults exhibit inadequate standing control in a narrow base of support or a disturbance of sensory information (Fujio and Takeuchi, 2021). In addition, elderly adults require higher descending neural input, even in natural standing, and may not have the sufficient reserve capacity to augment or change their descending neural input with the change in task characteristics. Thus, the flexibility of EMG-EMG coherence between standing tasks may reflect the ability of elderly adults to control standing balance and can be used as a biomarker to predict falls in future studies.

## Conclusion

The present review suggests that EMG-EMG coherence may help elucidate changes in standing control with age. Elderly adults have greater EMG-EMG coherence during natural bipedal standing than young adults, and significant coherence is observed over a broader frequency band. This could suggest that elderly adults require more descending neural input in bipedal standing and some cortical control of postural muscles. This analysis may contribute to understanding the mechanisms of balance disorders, not only age-related balance deficits, but also in individuals with CNS disorders, and should be investigated in a wider population. In addition, a consensus in the method of coherence analysis is required.

## Data availability statement

The original contributions presented in the study are included in the article/Supplementary material, further inquiries can be directed to the corresponding author.

## Author contributions

IN conceived the review paper idea and guided the literature and drafting of the manuscript. EY and YH conducted the literature search. EY prepared the manuscript for this review. All authors contributed to the article and approved the submitted version.
